# Fully-automated multi-organ segmentation tool applicable to both non-contrast and post-contrast abdominal CT: deep learning algorithm developed using dual-energy CT images

**DOI:** 10.1038/s41598-024-55137-y

**Published:** 2024-02-22

**Authors:** Sun Kyung Jeon, Ijin Joo, Junghoan Park, Jong-Min Kim, Sang Joon Park, Soon Ho Yoon

**Affiliations:** 1 Department of Radiology, Seoul National University Hospital, Seoul National University College of Medicine, 101 Daehak-ro, Jongno-gu, Seoul, 03080 Korea; 2 Department of Radiology, Seoul National University College of Medicine, Seoul, Korea; 3 Institute of Radiation Medicine, Seoul National University Medical Research Center Seoul National University Hospital, Seoul, Korea; 4 MEDICALIP. Co. Ltd., Seoul, Korea

**Keywords:** Medical research, Medical imaging

## Abstract

A novel 3D nnU-Net-based of algorithm was developed for fully-automated multi-organ segmentation in abdominal CT, applicable to both non-contrast and post-contrast images. The algorithm was trained using dual-energy CT (DECT)-obtained portal venous phase (PVP) and spatiotemporally-matched virtual non-contrast images, and tested using a single-energy (SE) CT dataset comprising PVP and true non-contrast (TNC) images. The algorithm showed robust accuracy in segmenting the liver, spleen, right kidney (RK), and left kidney (LK), with mean dice similarity coefficients (DSCs) exceeding 0.94 for each organ, regardless of contrast enhancement. However, pancreas segmentation demonstrated slightly lower performance with mean DSCs of around 0.8. In organ volume estimation, the algorithm demonstrated excellent agreement with ground-truth measurements for the liver, spleen, RK, and LK (intraclass correlation coefficients [ICCs] > 0.95); while the pancreas showed good agreements (ICC = 0.792 in SE-PVP, 0.840 in TNC). Accurate volume estimation within a 10% deviation from ground-truth was achieved in over 90% of cases involving the liver, spleen, RK, and LK. These findings indicate the efficacy of our 3D nnU-Net-based algorithm, developed using DECT images, which provides precise segmentation of the liver, spleen, and RK and LK in both non-contrast and post-contrast CT images, enabling reliable organ volumetry, albeit with relatively reduced performance for the pancreas.

## Introduction

Organ segmentation from medical images is garnering increasing attention owing to its potential clinical applications in fields such as computer-aided diagnosis, treatment planning, intraoperative assistance, and treatment delivery^1,2^. In abdominal CT scans, the volumes of solid abdominal organs acquired through organ segmentation are widely recognized for their diagnostic and prognostic significance. For instance, the liver-to-spleen volume ratio can be a useful parameter for evaluating portal hypertension^3^. Spleen volume predicts hepatocellular carcinoma occurrence and overall survival in patients with chronic liver disease^4^ and pancreatic volume is negatively associated with diabetes risk^5^. Additionally, total kidney volume influences the prognosis and treatment decisions for polycystic kidney disease^6^.

Traditionally, segmentation tasks have been performed manually by radiologists or with the assistance of semi-automated tools. However, the widespread adoption of manual or semi-automated methods in routine clinical practice is limited by the labor-intensive and time-consuming nature of the process^7^. Recent advancements in deep learning, specifically convolutional neural networks (CNNs), have addressed these limitations, leading to the development of various fully-automated segmentation methods. These methods have demonstrated high performance in certain organs, indicating their practical utility^8–10^. Most existing studies on deep-learning-based multi-organ segmentation from abdominal CT scans have primarily focused on algorithms designed for either non-contrast or post-contrast images^8–10^. However, given the variability in abdominal CT protocols in terms of dynamic phases, it would be beneficial to employ a unified algorithm that performs well across all image phases. 

In the development of deep learning-based segmentation algorithms applicable to non-contrast CT images, dual-energy CT (DECT) offers the advantage of providing reliable ground-truths for virtual non-contrast (VNC) images generated from post-contrast scans. Given their resemblance, VNC images could potentially be used as substitutes for true non-contrast (TNC) images for training purposes. By transferring the ground-truth from the corresponding post-contrast images to the VNC images, the algorithm can be trained precisely to ensure accurate organ segmentation. Prior studies have successfully employed this approach to develop segmentation algorithms for specific anatomic structures, such as pulmonary vessels^11^ and the heart^12^ in non-contrast images by incorporating VNC images into the training process. Expanding on this approach, the utilization of DECT extends beyond the development of algorithms for non-contrast images. By utilizing the paired VNC and post-contrast images sharing ground-truth information as training data, a unified algorithm that is applicable to both non-contrast and post-contrast images can be trained, offering a more efficient method for training data acquisition.

Therefore, this study aimed to develop a fully-automated algorithm for multi-organ segmentation of abdominal CT scans using dual-energy CT images applicable to both non-contrast and post-contrast images and to assess its accuracy in estimating organ volume.

## Methods

This study was approved by the Institutional Review Board of our institution, and the requirement for informed consent was waived because of the retrospective nature of the study.

### Data sources

To develop a multi-organ segmentation algorithm for abdominal CT scans, a dataset consisting of 95 DECT examinations was used which were randomly assigned into one of the three following sets: (1) training set (n = 75); (2) validation set (n = 10); and (3) training test set (n = 10). Furthermore, to evaluate the algorithm performance, two independent external datasets consisting of 30 DECT examinations (DECT test set) and 30 single-energy CT (SECT) examinations (SECT test set) were separately collected. Specific details of the datasets used in this study are provided below.

#### Dual-energy CT dataset

For the development of algorithm, we collected 95 dynamic contrast-enhanced abdominal CT examinations from 95 patients, conducted between April 2020 and September 2020, using two different DECT machines: SOMATOM Force, Siemens Healthineers (n = 49), and IQon Spectral CT, Philips Healthcare (n = 46). The inclusion criteria for the DECT dataset were generally healthy asymptomatic adult outpatients without focal lesions, with the exception of small cysts in the target organs, including the liver, spleen, right kidney (RK), left kidney (LK), and pancreas.

For external testing of algorithm performance, a temporally independent DECT test set consisting of 30 DECT examinations taken from January to February 2021 using two different DECT machines (SOMATOM Force, Siemens Healthineers [n = 15], and IQon Spectral CT, Philips Healthcare [n = 15]) was collected.

Dual-energy portal venous phase (DE-PVP) images were used as the dataset for post-contrast CT images. These DE-PVP images covered the region from the top of the higher hemidiaphragmatic dome to the anterior superior iliac spine or the upper thigh level. The CT acquisition and reconstruction parameters used for each scanner are presented in Supplementary Table 1. The corresponding VNC images were used as a dataset for non-contrast CT images. These VNC images were generated from the DE-PVP raw data using dedicated post-processing systems, such as Syngo.via for the SOMATOM Force and IntelliSpace Portal for IQon Spectral CT.

#### Single-energy CT dataset

For the SECT dataset, 30 dynamic contrast-enhanced abdominal CT examinations of 30 patients were collected (SECT test set), which were acquired using three different SECT scanners at our institution between January 2021 and February 2021: Revolution, GE Healthcare (n = 9), SOMATOM Definition, Siemens Healthineers (n = 11), and iCT, Philips Healthcare (n = 10). Each CT examination included TNC images and single-energy PVP (SE-PVP) images. The inclusion criteria for the SECT dataset were identical to those used for the DECT dataset. The scan range for both the SE-PVP and TNC was from the top of the higher hemidiaphragmatic dome down to the anterior superior iliac spine or upper thigh level. The detailed CT parameters for each scanner are listed in Supplementary Table 1.

### Creating 3D organ labels

3D organ labels for the liver, spleen, RK, LK, and pancreas were generated on both the DECT and SECT datasets. To improve the efficiency of the labeling process, a commercially available segmentation software (MEDIP PRO v.2.4.0, MEDICALIP Co. Ltd., Seoul, Korea) was initially used for preliminary organ segmentation. Subsequently, a board-certified abdominal radiologist (S.K.J., with 9 years of clinical experience in abdominal CT interpretation) manually performed voxel-wise correction of the preliminary labels, establishing them as ground truth annotations. For DECT, 3D organ labels were confirmed on DE-PVP images. These confirmed labels were then directly transferred to the corresponding VNC images based on spatiotemporal matching (Supplementary Fig. 1). In contrast, for SECT, organ labels were generated separately for the SE-PVP and TNC images. TNC labels were generated by referencing the PVP images of the same patient to ensure accuracy in the organ segmentation process.

### 3D nnU-Net algorithm for multi-organ segmentation

#### Algorithm development

**Figure 1:**
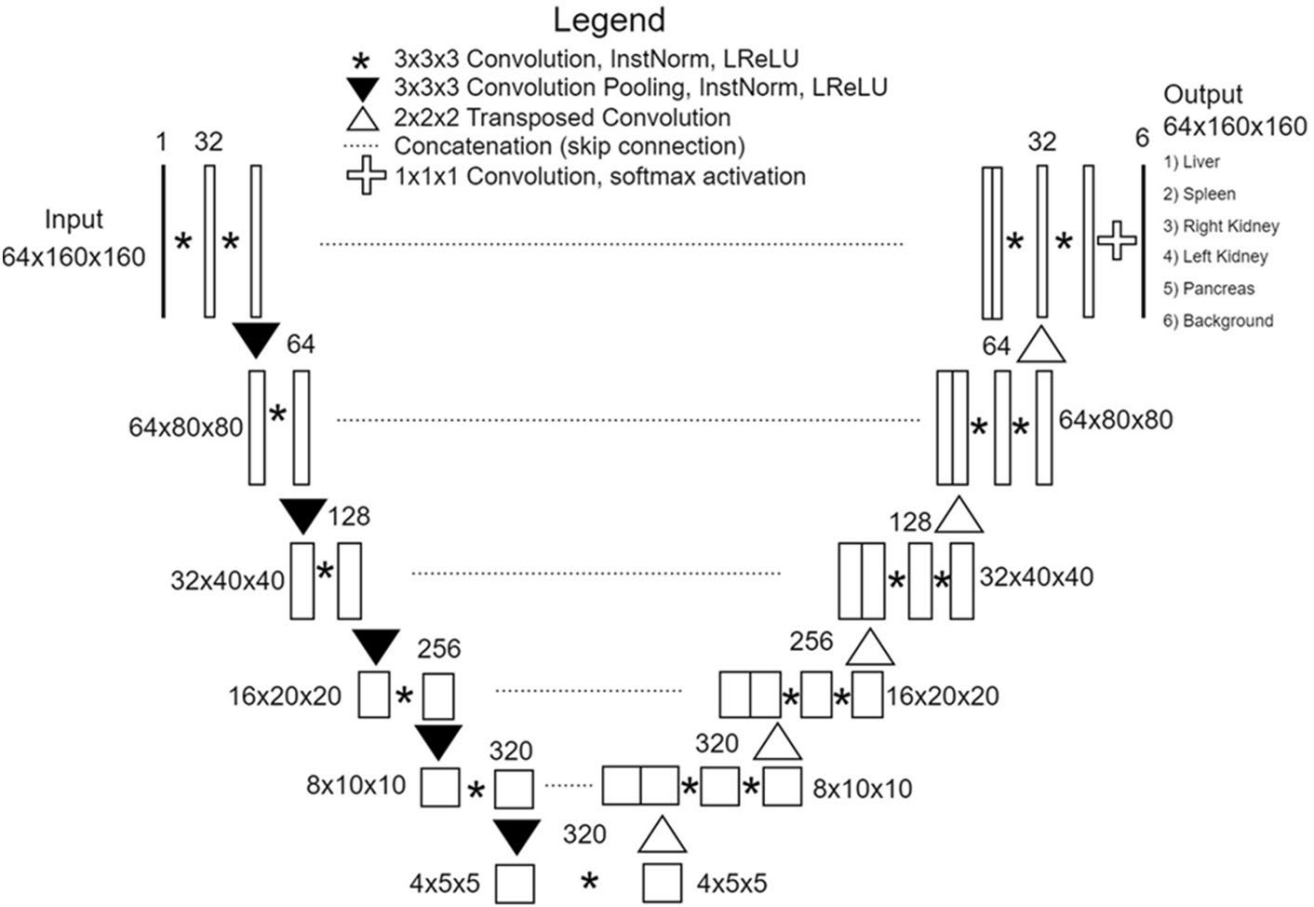
Architecture of 3D nnU-Net-based multi-organ segmentation algorithm.

A multi-organ segmentation algorithm for abdominal CT scans was developed using the 3D nnU-Net architecture (Fig. 1), which is a highly advanced CNN model known for its remarkable performance and efficacy in medical image segmentation^13^. To develop the algorithm, a dataset comprising 20,020 slices of PVP images and their corresponding VNC images from 85 DECT examinations with ground-truth annotations was used. Instead of training the segmentation algorithm end-to-end, the network was designed to take the internal organ areas predicted using a body composition segmentation algorithm^14^ as the input data, generating five classes corresponding to the segmented areas of the liver, spleen, RK, LK, and pancreas (Supplementary Fig. 2). The body composition segmentation algorithm we employed to predict internal organ areas operates by automatically providing volumetric segmentation of body components into seven classes: skin, bone, muscle, abdominal visceral fat, subcutaneous fat, central nervous system, and internal organs with vessels. Previous research reported dice similarity coefficients (DSCs) exceeding 0.94 for this algorithm^14^. We adopted this development strategy, using internal organ areas as input, to reduce inference time by strategically avoiding non-internal organ areas, consequently lowering computational load and reducing the occurrence of false positives.

Preprocessing strategies and network hyperparameters were customized to optimize the performance of the nnU-Net. As part of the preprocessing step, the 3D volumes were resized to match the target spacings of 0.66796875, 0.66796875, and 2. The final patch size configuration was 160 × 160 × 64, and the batch size was 2. During training, data augmentation techniques, such as rotation, scaling, gamma correction, and mirroring, were applied. Dice loss and cross-entropy loss functions were used to train the algorithm with a stochastic gradient descent (Nesterov momentum = 0.99). The polynomial learning rate scheduler is initialized to 0.01. The algorithm was trained for 1000 epochs.

#### Segmentation performance of the algorithm

First, the segmentation performance of the algorithm was evaluated using training test set, part of the development dataset not used for training. In addition, external tests were performed using two separated datasets (DECT test set and SECT test set). To evaluate the segmentation performance, both DE-PVP images and their corresponding VNC images were used in DECT set and both SE-PVP and TNC images were used for SECT set (Supplementary Fig. 3). 

### Organ volume estimation using the developed algorithm

The developed algorithm was tested for clinical applicability by evaluating its accuracy in measuring organ volumes in external test sets (DECT set and SECT set). The volume of each target organ (liver, spleen, RK, LK, and pancreas) was measured based on the 3D segmentation results obtained from the algorithm.

### Statistical analysis

The accuracy of the developed 3D nnU-net algorithm for organ segmentation was assessed for each organ and imaging phase by comparing the algorithm-derived masks with the ground truth masks. The dice similarity coefficient (DSC)^15^ was used as the evaluation metric. In addition, the DSC values of different imaging phases were compared between the DECT and SECT test sets. Specifically, the DSC values between the DE-PVP and VNC on DECT and between the SE-PVP and TNC on SECT were calculated using a paired *t*-test. Additionally, the DSC values were compared among difference CT machines using Mann–Whitney *U* test or Kruskal–Wallis test.

For organ volumetry, the correlation between the estimated volume obtained by the algorithm and the ground truth volume of each organ was assessed using Pearson correlation analysis. The agreement between the estimated and ground truth volumes was evaluated using the intraclass correlation coefficient (ICC) and Bland–Altman analysis. The ICC values were interpreted using the following criteria: ≥ 0.90 indicating excellent agreement; ≥ 0.75 to < 0.90, good agreement; ≥ 0.50 to < 0.75, moderate agreement; and < 0.50, poor agreement^16^. The Bland–Altman 95% limits of agreement (LOAs) were expressed as a percentage of the measured values. The percentage error of the algorithm-estimated volume was calculated by comparison with the ground-truth volume using the formula [(algorithm-estimated) − (ground truth)]/(ground truth) × 100 (%). Accurate volume estimation was defined as an assessment that deviated by no more than 10% of the ground truth volume. Accordingly, overestimation was defined as a deviation of > 10% of the ground truth volume, whereas underestimation was defined as a deviation of < − 10% of the ground truth volume.

All statistical analyses were performed using MedCalc version 19.4.0 (MedCalc Software, Ostend, Belgium). A *P* significance was set at < 0.05.

## Results

### Segmentation performance of the algorithm

#### Training test set

In test set, the developed algorithm achieved mean DSC values of 0.976, 0.963, 0.954, 0.954, and 0.879 for the liver, spleen, RK, LK, and pancreas in the DE-PVP images, and 0.974, 0.959, 0.945, 0.950, and 0.849 in the VNC images, respectively (Table 1).

**Table 1:** Dice similarity coefficients of the 3D nnU-Net-based algorithm in abdominal organ segmentation.

	Dice similarity coefficients (mean ± standard deviation)								
	Training test set			External test set—DECT			External test set—SECT		
	DE-PVP	VNC	P value*	DE-PVP	VNC	P value*	SE-PVP	TNC	P value*
Liver	0.976 ± 0.004	0.973 ± 0.007	0.206	0.986 ± 0.003	0.977 ± 0.006	< 0.001	0.981 ± 0.005	0.965 ± 0.012	< 0.001
Spleen	0.963 ± 0.012	0.959 ± 0.017	0.280	0.978 ± 0.011	0.966 ± 0.016	< 0.001	0.972 ± 0.015	0.961 ± 0.019	< 0.001
Right kidney	0.954 ± 0.017	0.945 ± 0.018	< 0.001	0.976 ± 0.006	0.968 ± 0.008	< 0.001	0.971 ± 0.007	0.944 ± 0.016	< 0.001
Left kidney	0.955 ± 0.015	0.950 ± 0.018	0.135	0.976 ± 0.007	0.970 ± 0.008	< 0.001	0.970 ± 0.007	0.954 ± 0.011	< 0.001
Pancreas	0.879 ± 0.045	0.849 ± 0.046	0.001	0.873 ± 0.045	0.854 ± 0.046	< 0.001	0.846 ± 0.060	0.810 ± 0.045	< 0.001

*DECT* dual-energy CT, *SECT* single-energy CT, *DE-PVP* portal venous phase on dual-energy CT, *VNC* virtual non-contrast, *SE-PVP* portal venous phase on single-energy CT, *TNC* true non-contrast. *P-values were calculated using a paired *t*-test (DE-PVP vs. VNC and SE-PVP vs. TNC).

#### External test set

In the external DECT test set, the developed algorithm achieved mean DSC values of 0.986, 0.978, 0.976, 0.976, and 0.873 for the liver, spleen, RK, LK, and pancreas in the DE-PVP images, and 0.977, 0.966, 0.968, 0.970, and 0.854 in the VNC images, respectively (Table 1). All target organs showed significantly higher DSC values in the DE-PVP images than in the VNC images (all Ps < 0.001). Mean DSC values were not significantly different based on CT machines for all target organs (Ps > 0.05, Supplementary Table 2). In the external SECT test set, the mean DSC values of the developed algorithm were 0.981, 0.972, 0.971, 0.970, and 0.846 for the liver, spleen, RK, LK, and pancreas in the SE-PVP images and 0.965, 0.961, 0.944, 0.954, and 0.810 in the TNC images, respectively (Table 1). The DSC values of all target organs were significantly higher in the SE-PVP images than in the TNC images (all Ps < 0.001). Mean DSC values were not significantly different based on CT machines for all target organs (Ps > 0.05, Supplementary Table 2).

### Application for organ volumetry

#### DECT test set

In the DECT test set, the algorithm-estimated volume and ground-truth volume of all target organs showed strong correlations in both DE-PVP and VNC images (Pearson’s r = 0.999, 0.999, 0.994, 0.990, and 0.917 in DE-PVP; and 0.999, 0.999, 0.993, 0.987, and 0.946 in VNC, for the liver, spleen, RK, LK, and pancreas, respectively; all Ps < 0.001). The algorithm-estimated volume showed excellent agreement with the ground truth for the liver, spleen, RK, and LK (all ICCs > 0.9) in both the DE-PVP and VNC images. However, for the pancreas, the agreement was good, with ICC values of 0.889 and 0.890 for DE-PVP and VNC, respectively (Table 2). The algorithm-estimated volume of the liver, spleen, RK, LK, and pancreas showed mean biases with 95% LOAs in comparison to the ground truth volume as follows: − 0.4% (− 1.9% to 1.1%), 0% (− 4.3% to 4.3%), − 1.6% (− 5.3% to 2.1%), − 2.0% (− 6.2% to 2.2%), and 6.8% (− 14.7% to 28.4%) in DE-PVP images (Supplementary Fig. 4); and 0.2% (− 2.2% to 2.6%), − 0.2% (− 5.2% to 4.9%), − 0.6% (− 5.0% to 3.8%), − 1.7% (− 6.7% to 3.3%), and 9.2% (− 8.5% to 26.9%) in VNC images, respectively (Supplementary Fig. 5).

**Table 2:** Agreements between algorithm-estimated volume and ground-truth volume of each organ.

	Intraclass correlation coefficient (95% confidence interval)					
	Training test set		External test set—DECT		External test set—SECT	
	DE-PVP	VNC	DE-PVP	VNC	SE-PVP	TNC
Liver	0.999 (0.995, 0.999)	0.999 (0.997, 0.999)	0.999 (0.998, 0.999)	0.999 (0.997, 0.999)	0.998 (0.996, 0.999)	0.985 (0.772, 0.996)
Spleen	0.999 (0.996, 0.999)	0.999 (0.996, 0.999)	0.999 (0.998, 0.999)	0.999 (0.997, 0.999)	0.999 (0.998, 0.999)	0.992 (0.984, 0.996)
Right kidney	0.925 (0.697, 0.981)	0.916 (0.660, 0.979)	0.991 (0.957, 0.997)	0.992 (0.984, 0.996)	0.989 (0.961, 0.996)	0.971 (0.816, 0.991)
Left kidney	0.927 (0.704, 0.982)	0.914 (0.653, 0.979)	0.983 (0.884, 0.995)	0.981 (0.935, 0.993)	0.982 (0.839, 0.994)	0.970 (0.454, 0.993)
Pancreas	0.892 (0.566, 0.973)	0.887 (0.546, 0.972)	0.889 (0.689, 0.954)	0.890 (0.395, 0.965)	0.792 (0.231, 0.926)	0.840 (0.694, 0.920)

*DECT* dual-energy CT, *SECT* single-energy CT, *DE-PVP* portal venous phase on dual-energy CT, *VNC* virtual non-contrast, *SE-PVP* portal venous phase on single-energy CT, *TNC* true non-contrast.

The algorithm achieved accurate volume estimation, deviating within 10% from ground-truth, for the liver, spleen, RK, and LK volumes in all assessed cases (100%) in both the DE-PVP and VNC images. However, for the pancreas, the percentage of accurate volume estimation was lower, with 56.7% (17/30) in the DE-PVP images and 60.0% (18/30) in the VNC images (Supplementary Table 3).

#### SECT test set

**Figure 2:**
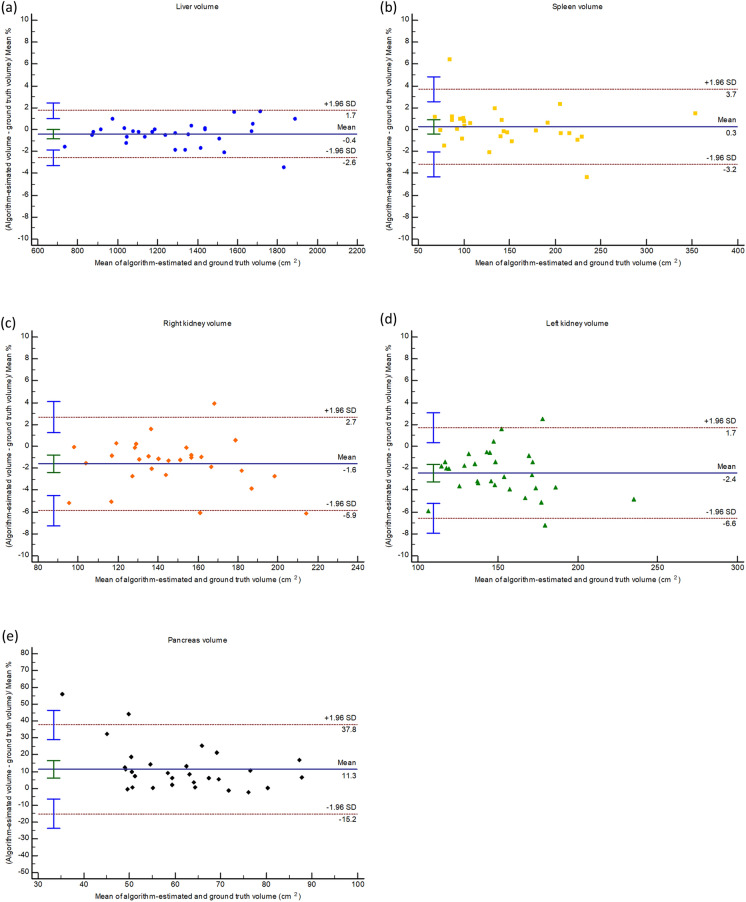
Bland–Altman plots illustrating the agreement between algorithm-estimated volumes and ground-truth volumes of the (**a**) liver, (**b**) spleen, (**c**) right kidney, (**d**) left kidney, and (**e**) pancreas in the external single-energy CT test set’s portal venous phase images.

**Figure 3:**
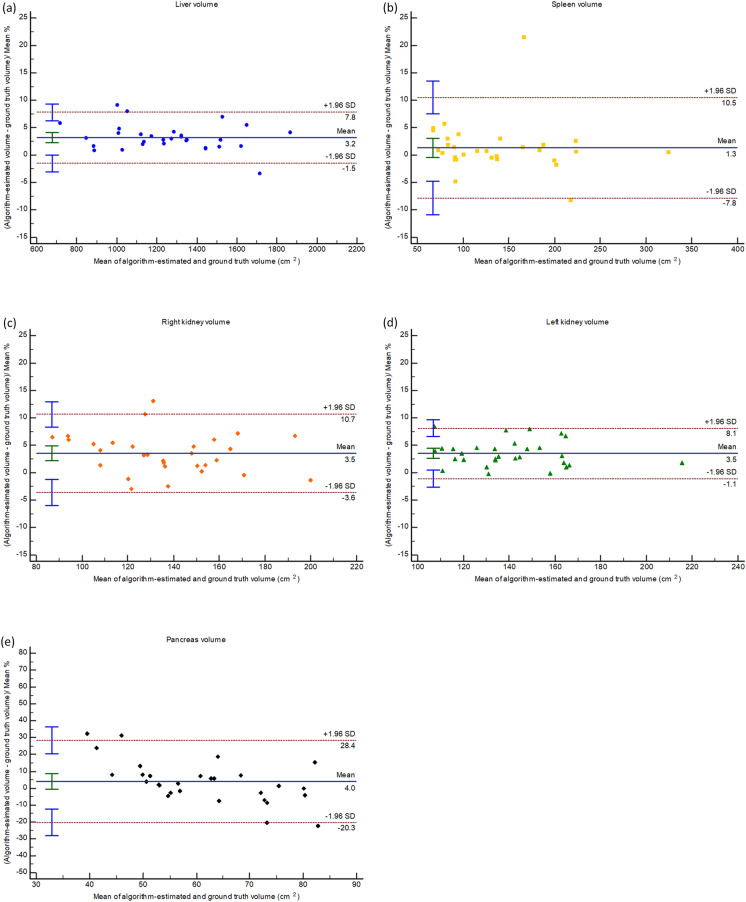
Bland–Altman plots illustrating the agreement between algorithm-estimated volumes and ground-truth volumes of the (**a**) liver, (**b**) spleen, (**c**) right kidney, (**d**) left kidney, and (**e**) pancreas in the external single-energy CT test set’s true non-contrast images.

In the SECT test set, the algorithm-estimated volume and ground-truth volume of all target organs showed strong correlations in both SE-PVP and TNC images (r = 0.998, 0.999, 0.993, 0.992, and 0.883 in SE-PVP; and 0.994, 0.992, 0.984, 0.990, and 0.877 in TNC for the liver, spleen, RK, LK, and pancreas, respectively; all Ps < 0.001). The algorithm-estimated volume showed excellent agreement with the ground truth for the liver, spleen, RK, and LK (all ICCs > 0.9) in both the SE-PVP and TNC images. However, the agreement was good for the pancreas, with ICC values of 0.792 and 0.840 for SE-PVP and TNC, respectively (Table 2). The algorithm-estimated volume of the liver, spleen, RK, LK, and pancreas showed mean biases with 95% LOAs in comparison to the ground-truth volume as follows: − 0.4% (− 2.6% to 1.7%), 0.3% (− 3.2% to 3.7%), − 1.6% (− 5.9% to 2.7%), − 2.4% (− 6.6% to 1.7%), and 11.3% (− 15.2% to 37.8%) in SE-PVP images (Fig. 2); and 3.2% (− 1.5% to 7.8%), 1.3% (− 7.8% to 10.5%), 3.5% (− 3.6% to 10.7%), 3.5% (− 1.1% to 8.1%), and 4.0% (− 20.3% to 28.4%) in TNC images (Fig. 3).

The algorithm achieved accurate volume estimation deviating within 10% from ground-truth for the liver, spleen, RK, and LK volumes in over 90% of the cases in both SE-PVP and TNC images. However, for the pancreas, the percentage of accurate volume estimation was lower, with 56.7% (17/30) in the SE-PVP images and 73.3% (22/30) in the TNC images (Supplementary Table 3 and Supplementary Fig. 6).

## Discussion

In this study, we successfully developed a fully-automated algorithm for multi-organ segmentation in abdominal CT scans that is applicable to both non-contrast and post-contrast imaging. The algorithm was efficiently developed by utilizing PVP (post-contrast) images and their spatiotemporally-matched VNC (non-contrast) images from DECT scans, thereby enabling the sharing of organ masks from PVP to VNC images during the development process. The developed algorithm was validated using both DECT and SECT scans (external tests) to assess its general usability. The results of our 3D nnU-Net algorithm showed highly accurate segmentation of the liver, spleen, RK, and LK in abdominal CT scans, regardless of contrast enhancement, with mean DSCs exceeding 0.96 in the DECT test set and exceeding 0.94 in the SECT test set. The versatility of our algorithm in terms of imaging phases may allow data collection with diverse CT protocols, thus enhancing the clinical utility of the organ segmentation results. However, for pancreatic segmentation, the algorithm demonstrated a relatively reduced performance, with a mean DSC ranging from 0.810 to 0.873 in the external test sets.

Recent studies have reported promising results for the automated segmentation of abdominal organs from abdominal CT scans using various deep learning methods^3,10,17–24^. These algorithms demonstrate good segmentation performance, particularly in post-contrast CT imaging. The reported mean DSCs ranged from 0.93 to 0.97 for the liver^10,17–19^, from 0.92 to 0.96 for the spleen^22,25–27^, and from 0.86 to 0.97 for the kidney^20,21,28^. Our algorithm achieved excellent results on the external validation set for post-contrast imaging, with DSC values of 0.981, 0.972, 0.971, and 0.970 for the liver, spleen, RK, and LK, respectively, which were similar to the best results reported in previous studies for each organ. However, there have been limited research on non-contrast CT imaging, which is a more challenging task for organ segmentation^29^. Previous studies using non-contrast CT reported relatively lower segmentation performance compared with post-contrast CT, with the mean DSC of 0.86–0.95^23,24^ for the liver and 0.62 for spleen^22^. However, our study achieved reliable results even with non-contrast CT imaging, as evidenced by the mean DSC values of 0.965, 0.961, 0.944, and 0.954 for the liver, spleen, RK, and LK, respectively, in the TNC images of the external validation set. Although these values were lower than the post-contrast performance of our algorithm, they still indicated excellent segmentation accuracy. These encouraging results can be attributed to the successful incorporation of VNC images in the development of the algorithm, which facilitated precise segmentation mask generation in non-contrast CT images in conjunction with the implementation of the advanced 3D nnU-Net methodology. Segmentation performance of pancreas has been reported to be lower (mean DSC, 0.63–0.87)^24,30^ than that of other abdominal solid organs, which is in agreement with our study results. The reasons for this can be attributed to the inherent constraints of the pancreas, including its lobulated shape and the difficulty in distinguishing it from nearby structures, such as the collapsed duodenum or small bowel, and lymph nodes, even for experienced radiologists^31,32^.

Our algorithm can estimate organ volumes based on 3D segmentation results, demonstrating excellent agreement (with ICCs > 0.9) with ground-truth volumes for the liver, spleen, RK, and LK in the external test sets. It successfully achieved accurate volume estimation, deviating within 10% of the ground-truth in over 90% of the cases for these organs. This can be primarily attributed to the precise segmentation performance of the algorithm. Given the significant interest in organ volumetry, our automated algorithm holds excellent value for various diagnostic and prognostic applications in both research and clinical practice. Our results for organ volumetry are in line with those of previous studies that also reported good performance in the liver, spleen, and kidneys^3,33–35^. The pancreas, as inferred from its comparatively lower segmentation performance, exhibited a slightly lower agreement with the ground-truth, as indicated by ICCs ranging from 0.792 to 0.890. The accurate volume estimation rate of the pancreas ranged from 56.7 to 73.3% in the external test set. In our study, underestimation of the pancreatic volume frequently resulted from omitting the terminal portions of the head or tail of the pancreas or additional lobulations of pancreatic tissue, whereas overestimation often arose from including the iso-attenuating adjacent duodenum or collapsed jejunum. Overcoming these challenges would be crucial for improving segmentation performance of the pancreas.

Our study had several limitations. First, because our development and external test sets primarily comprised cases with minimal segmentation challenges (specifically, patients without focal lesions, except for small cysts in abdominal solid organs), the generalizability of our algorithm needs to be further evaluated for patients with diverse diseases. Second, although our external test set encompassed three different single-energy CT scanners with non-contrast and post-contrast images, additional validation is required involving various CT scanners, protocols, and phases of dynamic imaging. Third, we did not investigate the practical value of our algorithm in a clinical setting. Further studies are necessary to evaluate its real-world applicability for specific clinical applications.

In conclusion, our 3D nnU-Net-based algorithm, developed using DECT images, accurately segmented abdominal solid organs on both non-contrast and post-contrast CT images, enabling reliable organ volumetry of the liver, spleen, and right and left kidneys, albeit with relatively lower performance for the pancreas.

### Supplementary Information

Supplementary Information.

## Data Availability

The data used to support the findings of this study are available from the corresponding author on reasonable request.

## Code availability

The artificial intelligence algorithm developed from this study is available through the commercial product (MEDIP PRO, MEDICALIP Co. Ltd., Seoul, Korea) and the code can be available from the corresponding author on reasonable request.
