# Supplementary material for: Fully-automated multi-organ segmentation tool applicable to both non-contrast and post-contrast abdominal CT: deep learning algorithm developed using dual-energy CT images
Source: Sci Rep. 2024 Feb 22;14:4378. doi: 10.1038/s41598-024-55137-y (PMC10883917; doi:10.1038/s41598-024-55137-y)

**Fully-automated multi-organ segmentation tool applicable to both non-contrast and post-contrast abdominal CT: deep learning algorithm developed using dual-energy CT images**

Manuscript type: Original Research

Sun Kyung Jeon, M.D.^1, 2^, *Ijin Joo, M.D.^1, 2, 3^, Junghoan Park, M.D.^1, 2^, Jong-Min Kim, Ph.D.^4^, Sang Joon Park, Ph.D.^4^, Soon Ho Yoon, M.D.^1,2,4^

^1^Department of Radiology, Seoul National University Hospital, ^2^Department of Radiology, Seoul National University College of Medicine, ^3^Institute of Radiation Medicine, Seoul National University Medical Research Center Seoul National University Hospital, ^4^MEDICALIP. Co. Ltd., Seoul, Korea

**Corresponding author: Ijin Joo, M.D.**

Department of Radiology, Seoul National University Hospital, Department of Radiology, Seoul National University College of Medicine, 101 Daehak-ro, Jongno-gu, Seoul, 03080, Korea

TEL: (822) 2072-3107 FAX: (822) 743-6385   E-mail: hijijin@gmail.com

**Supplementary Table 1.** CT acquisition parameters

| CT scanner | SOMATOM Force | IQon Spectral CT | Revolution | SOMATOM Definition | iCT |
| --- | --- | --- | --- | --- | --- |
| Scanner type | Dual-source Scanner | Single-source scanner with dual-layer detector | Single-energy scanner | Single-energy scanner | Single-energy scanner |
| Tube voltage (kVp) | 80 and 150 | 120 | 80 | 100 | 120 |
| Effective mAs | 91-246 | 43-173 | 249-350 | 117-303 | 96-139 |
| Slice thickness (mm) | 3 | 3 | 2.5 | 3 | 3 |
| Matrix | 512 x 512 | 512 x 512 | 512 x 512 | 512 x 512 | 512 x 512 |
| Gantry rotation time (s) | 0.5 | 0.33 | 0.5 | 0.5 | 0.4 |
| Detector pitch | 0.6 | 0.797 | 0.992 | 1 | 0.507 |

**Supplementary Table 2.** Dice similarity coefficients of the 3D nnU-Net-based algorithm in abdominal organ segmentation based on CT machines

|  | Dice similarity coefficient (mean ± standard deviation) | | | | | | | | | | | |
| --- | --- | --- | --- | --- | --- | --- | --- | --- | --- | --- | --- | --- |
| **DECT test set** | DE-PVP | | | | | | VNC | | | | | |
|  | SOMATOM Force | | IQon Spectral CT | | P value^*^ | | SOMATOM Force | | IQon Spectral CT | | P value^*^ | |
| Liver | 0.985 ± 0.003 | | 0.986 ± 0.003 | | 0.663 | | 0.976 ± 0.006 | | 0.979 ± 0.006 | | 0.678 | |
| Spleen | 0.978 ± 0.010 | | 0.978 ± 0.012 | | 0.983 | | 0.966 ± 0.014 | | 0.965 ± 0.019 | | 0.917 | |
| Right kidney | 0.977 ± 0.005 | | 0.976 ± 0.007 | | 0.431 | | 0.970 ± 0.006 | | 0.967 ± 0.010 | | 0.221 | |
| Left kidney | 0.976 ± 0.007 | | 0.977 ± 0.006 | | >0.999 | | 0.967 ± 0.008 | | 0.971 ± 0.008 | | 0.547 | |
| Pancreas | 0.856 ± 0.046 | | 0.890 ± 0.038 | | 0.059 | | 0.843 ± 0.042 | | 0.866 ±0.047 | | 0.604 | |
| **SECT test set** | SE-PVP | | | | | | TNC | | | | | |
|  | Revolution | Definition | | iCT | | P value^§^ | Revolution | Definition | | iCT | | P value^§^ |
| Liver | 0.980 ± 0.005 | 0.984 ± 0.005 | | 0.980 ± 0.005 | | 0.051 | 0.967 ± 0.018 | 0.963 ± 0.012 | | 0.968 ±0.005 | | 0.218 |
| Spleen | 0.972 ± 0.008 | 0.970 ± 0.024 | | 0.974 ± 0.006 | | 0.434 | 0.965 ± 0.018 | 0.963 ± 0.012 | | 0.968 ± 0.005 | | 0.602 |
| Right kidney | 0.968 ±0.007 | 0.977 ± 0.005 | | 0.967 ± 0.005 | | 0.200 | 0.944 ± 0.011 | 0.945 ± 0.018 | | 0.942 ± 0.018 | | 0.830 |
| Left kidney | 0.967 ± 0.008 | 0.974 ± 0.007 | | 0.969 ± 0.005 | | 0.158 | 0.955 ± 0.010 | 0.952 ± 0.012 | | 0.956 ± 0.010 | | 0.628 |
| Pancreas | 0.856 ± 0.042 | 0.829 ±0.089 | | 0.855 ±0.042 | | 0.895 | 0.818 ± 0.045 | 0.793 ± 0.052 | | 0.821 ± 0.035 | | 0.476 |

DECT, dual-energy CT; SECT, single-energy CT; DE-PVP, portal venous phase on dual-energy CT; VNC, virtual non-contrast; SE-PVP, portal venous phase on single-energy CT; TNC, true non-contrast. ^*^P-values were calculated using Mann-Whitney U test. ^§^P-values were calculated using Kruskal-Wallis test\

**Supplementary Table 3.** Performance of the algorithm for organ volume estimation

|  | Accurate estimation* | Under-estimation | Over-estimation |
| --- | --- | --- | --- |
| **DECT test set (n, %)** | | | |
| DE-PVP |  |  |  |
| Liver | 30 (100) | 0 (0) | 0 (0) |
| Spleen | 30 (100) | 0 (0) | 0 (0) |
| Right kidney | 30 (100) | 0 (0) | 0 (0) |
| Left kidney | 30 (100) | 0 (0) | 0 (0) |
| Pancreas | 17 (56.7) | 0 (0) | 13 (43.3) |
| VNC |  |  |  |
| Liver | 30 (100) | 0 (0) | 0 (0) |
| Spleen | 30 (100) | 0 (0) | 0 (0) |
| Right kidney | 30 (100) | 0 (0) | 0 (0) |
| Left kidney | 30 (100) | 0 (0) | 0 (0) |
| Pancreas | 18 (60.0) | 1 (3.3) | 11 (36.7) |
| **SECT test set (n, %)** | | | |
| SE-PVP |  |  |  |
| Liver | 30 (100) | 0 (0) | 0 (0) |
| Spleen | 30 (100) | 0 (0) | 0 (0) |
| Right kidney | 30 (100) | 0 (0) | 0 (0) |
| Left kidney | 30 (100) | 0 (0) | 0 (0) |
| Pancreas | 17 (56.7) | 0 (0) | 13 (43.3) |
| TNC |  |  |  |
| Liver | 30 (100) | 0 (0) | 0 (0) |
| Spleen | 29 (96.7) | 1 (3.3) | 0 (0) |
| Right kidney | 28 (93.3) | 0 (0) | 2 (6.7) |
| Left kidney | 30 (100) | 0 (0) | 0 (0) |
| Pancreas | 22 (73.3) | 2 (6.7) | 6 (20.0) |

DE-PVP = portal venous phase image of dual-energy CT, VNC = virtual non-contrast, SE-PVP = portal venous phase image of single-energy CT, TNC = true non-contrast. *Accurate estimation was defined as assessment within a deviation of 10% of ground-truth volume. Over-estimation and under-estimation were defined as >10% and <-10% of ground-truth volume, respectively.

**Supplementary Figure 1.** Example case demonstrating the 3D organ label transfer from portal venous phase to corresponding virtual non-contrast imaging for ground truth organ mask generation. In a 39-year-old male, the DECT PVP image (a) and its spatiotemporally matched VNC image (b) form a pair. Leveraging this matching, direct transfer of organ labels from the PVP image (c) to the corresponding VNC image (d) enables accurate organ segmentation.

**
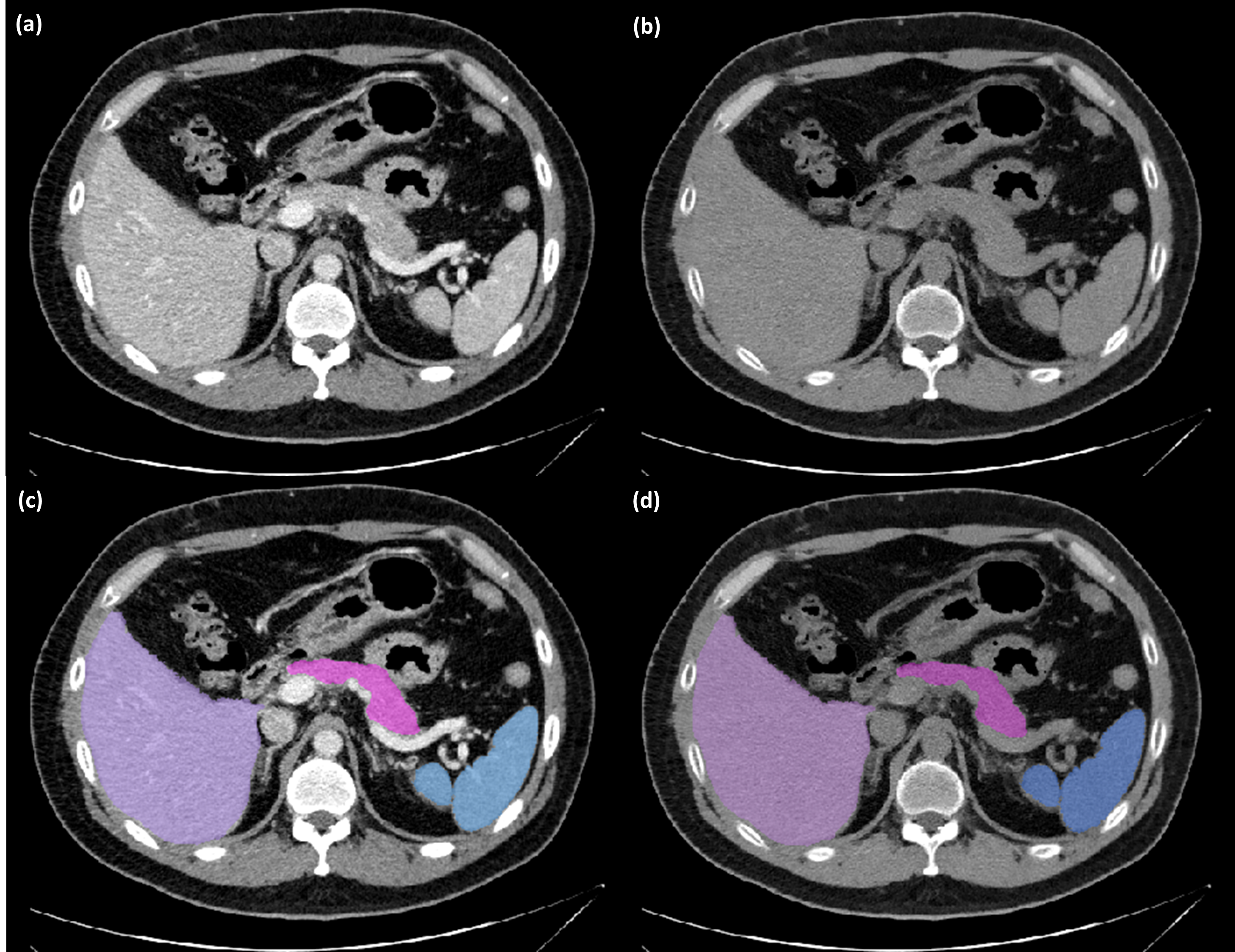
**

**Supplementary Figure 2.** Illustration of the input and output data utilized in our 3D nnU-Net-based algorithm development. The algorithm was designed to take the volumetric internal organ areas as the input data (a: axial image, b: coronal image) and output five classes corresponding to the segmented areas of the liver, spleen, RK, LK, and pancreas (c)

**
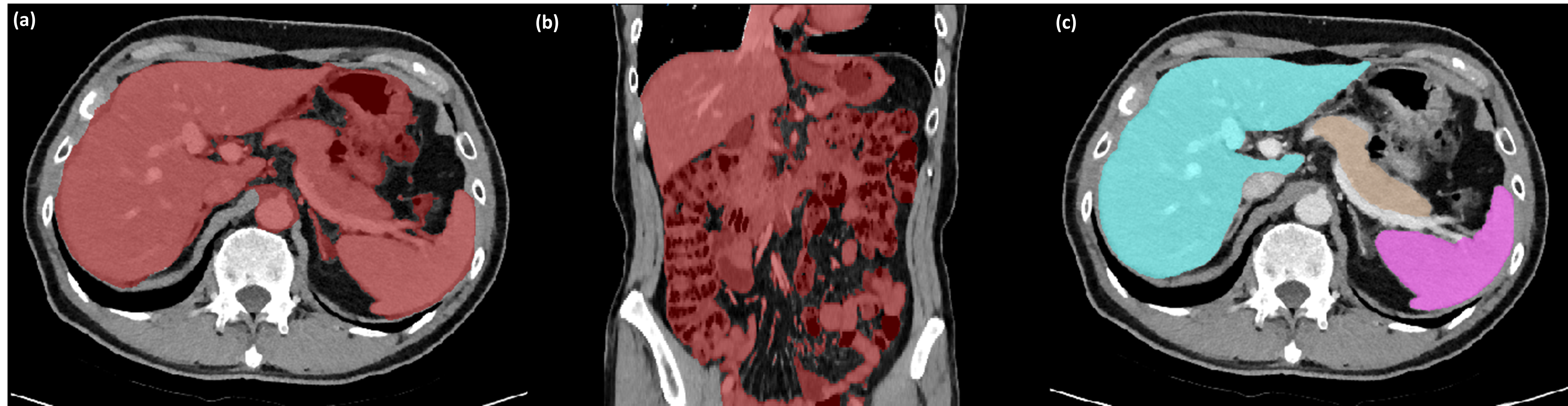
**

**Supplementary Figure 3.** Illustration of fully-automated multi-organ segmentation outcomes using the 3D nnU-Net-based algorithm. The segmentation process involves the liver, spleen, right kidney, left kidney, and pancreas on single-energy abdominal CT scans of a 42-year-old male. (a, b) 3D volume rendering image (a) and an axial image (b) from the portal venous phase, respectively; (c) an axial image from the true non-contrast phase.


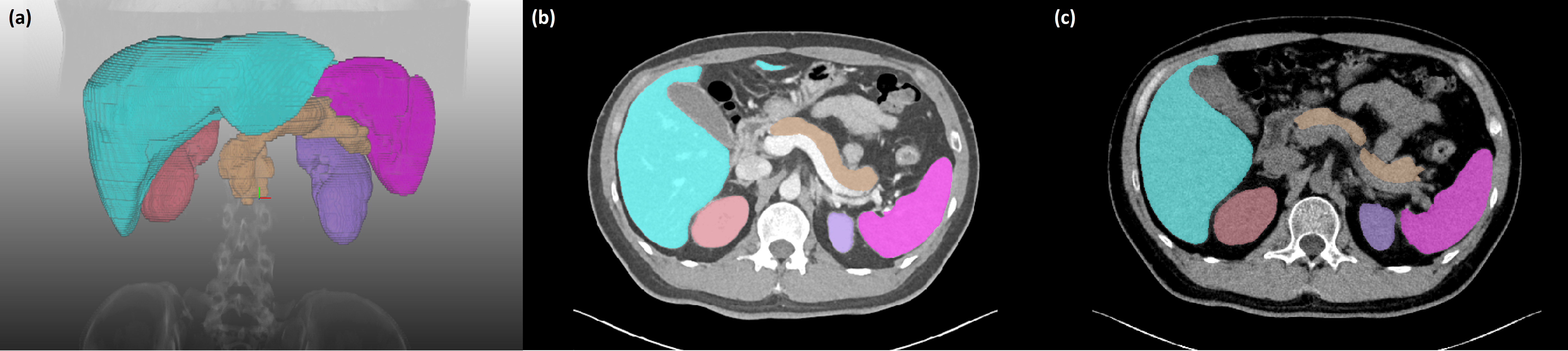


**Supplementary Figure 4.** Bland-Altman plots illustrating the agreement between algorithm-estimated volumes and ground truth volumes of the (a) liver, (b) spleen, (c) right kidney, (d) left kidney, and (e) pancreas in the dual-energy CT test set’s portal venous phase images.

**
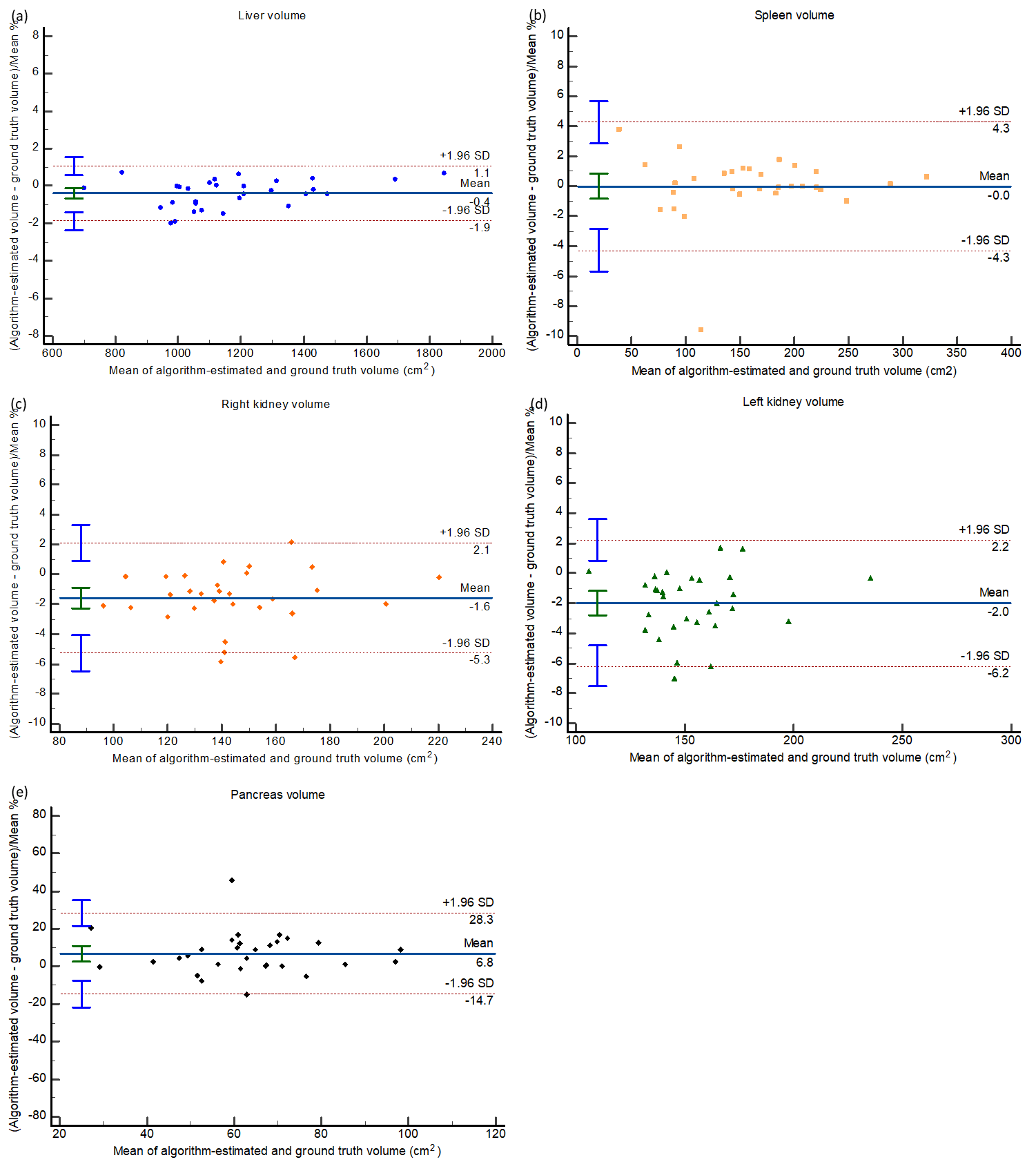
**

**Supplementary Figure 5.** Bland-Altman plots illustrating the agreement between algorithm-estimated volumes and ground truth volumes of the (a) liver, (b) spleen, (c) right kidney, (d) left kidney, and (e) pancreas in the dual-energy CT test set’s virtual non-contrast images.

**
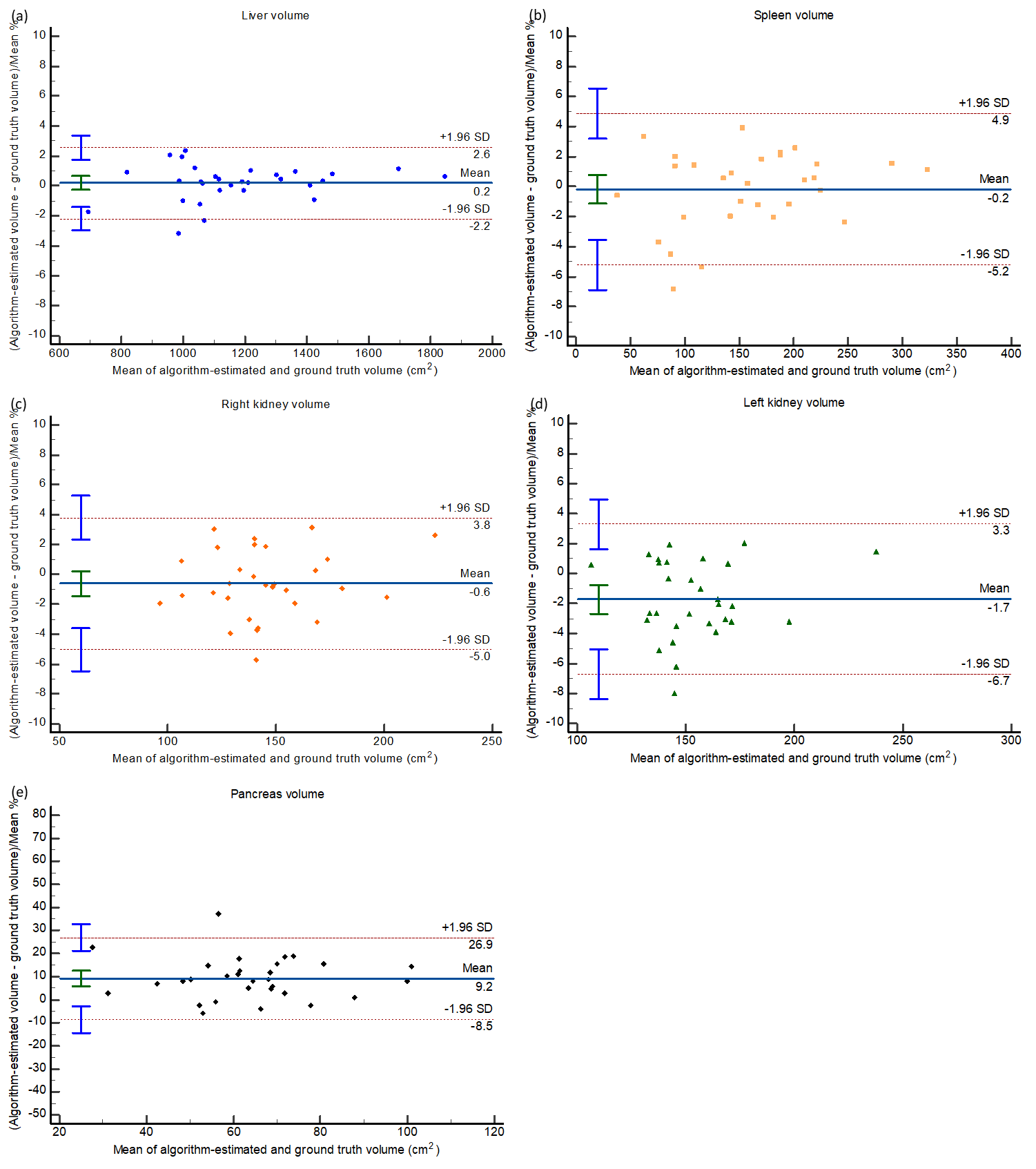
**

**Supplementary Figure 6.** Illustration of segmentation results demonstrating underestimation (a,b) and overestimation (c,d) of pancreatic volume. (a, b) An example case of underestimation of the pancreatic volume resulting from omitting the terminal portions of the head of the pancreas (arrows), and (c, d) another case of overestimation resulting from including iso-attenuating adjacent gastric fold and collapsed small bowel (arrows).


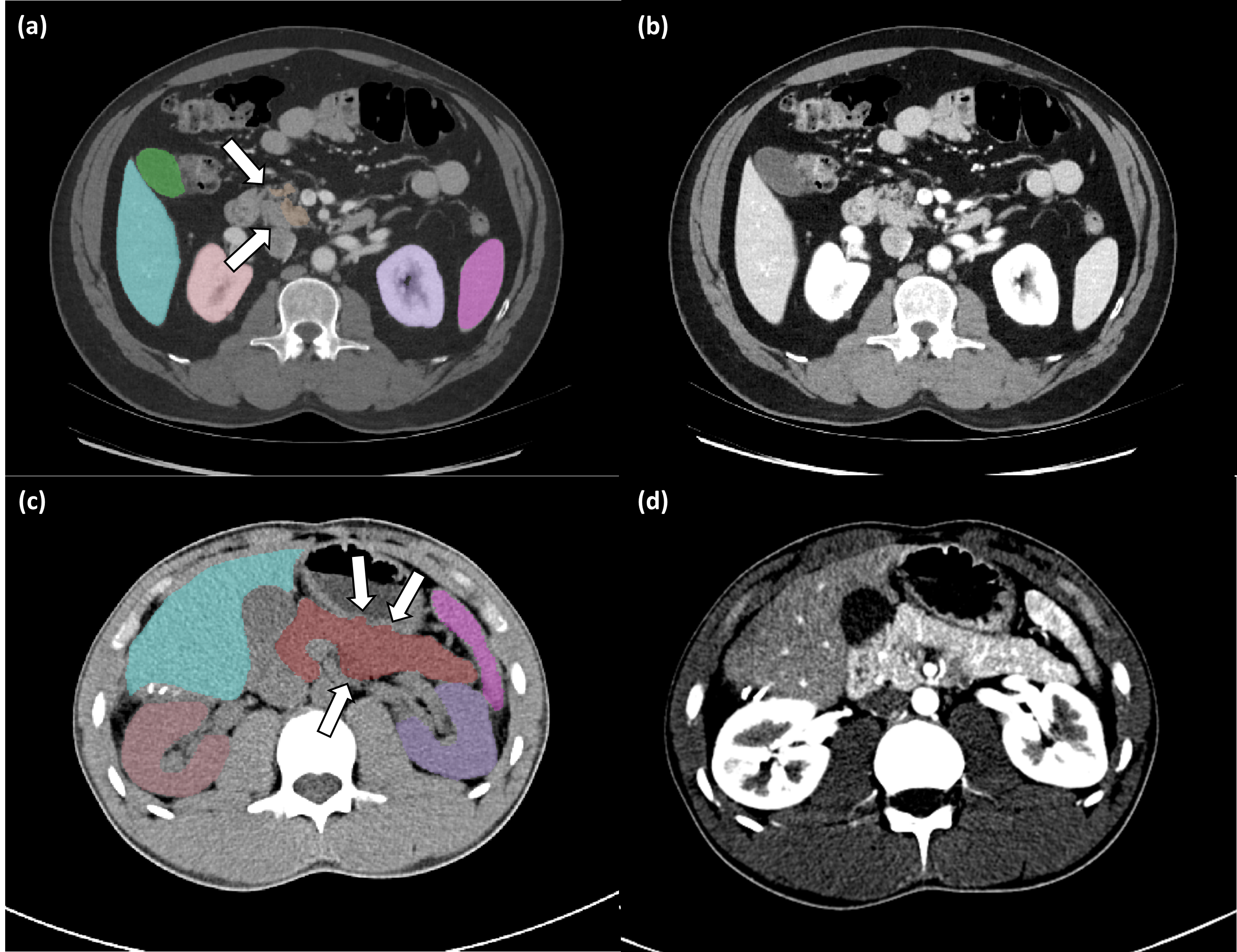

Supplement: Supplementary file 1 — Supplementary Information. [file 41598_2024_55137_MOESM1_ESM.docx]
